# Supplementary material for: Long term trends in NHS inpatient bed provision in England, 1960–2020
Source: PLoS One. 2025 Aug 29;20(8):e0330931. doi: 10.1371/journal.pone.0330931 (PMC12396719; doi:10.1371/journal.pone.0330931)

**Supplementary Table S1. The number of five different categories of NHS beds and the provision of beds per 100,000 population in England in 1960 and 2019/20.**

| NHS bed category | Number of available inpatient beds.  ***Provision of NHS beds per 100,000 population.*** | | Change in Number of beds  ***Change in provision of beds per 100,000*** | Percentage reduction in beds  ***Percentage reduction in provision*** |
| --- | --- | --- | --- | --- |
|  | 1960 | 2019/20 |  |  |
| Acute | 172,584  ***400*** | 83,584  ***148*** | 89,000  ***252*** | 51.6%  ***63.0%*** |
| Geriatric | 55,146  ***128***  ***1,081**** | 17,848  ***32***  ***173**** | 37,298  ***96***  ***911**** | 67.6%  ***75.0%***  ***84.3%**** |
| Maternity | 18,513  ***43*** | 7,666  ***14*** | 10,844  ***29*** | 58.7%  ***67.4%*** |
| Mental Illness | 144,072  ***334*** | 18,171  ***32*** | 125,901  ***302*** | 87.4 %  ***90.6%*** |
| Learning Disability | 57,204  ***132*** | 947  ***2*** | 56,255  ***132*** | 98.3 %  ***98.7 %*** |

* Rates for Geriatric beds calculated per 100,000 of the population over the age of 65 years. All other rates are per 100,000 of the total population.

The population estimates used were as follows: Total population of England was 43,188,713 in 1960 with 5,101,388 aged over the age of 65 years. Total population in England was 56,286,853 in 2019/20 with 10,468,953 over the age of 65 years.

**Supplementary Table S2. Model coefficients from a restricted cubic spline fit (7 knots) on annual percentage change in NHS patient bed numbers (overall) vs year between 1960 and 2020**

| **Coefficient** | **Estimate (Std error)** | **P-value** |
| --- | --- | --- |
| (Intercept) | 262.40 (232.21) | 0.260 |
| rcs(Year, 7)Year | -0.13 (0.12) | 0.260 |
| rcs(Year, 7)Year' | 3.83 (2.30) | 0.100 |
| rcs(Year, 7)Year'' | -13.24 (5.62) | 0.020 |
| rcs(Year, 7)Year''' | 26.35 (6.72) | <0.001 |
| rcs(Year, 7)Year'''' | -36.69 (6.88) | <0.001 |
| rcs(Year, 7)Year''''' | 36.45 (6.72) | <0.001 |
| R-squared: 0.5105  Model p-value: <0.0001 | | |

Supplementary Figure S1. The ratio of annual number of deaths to NHS General & Acute inpatient beds (solid line and left axis) and the ratio of births to NHS Maternity beds (dashed line and right axis). Data refers to England between 1960 - 2019/20.


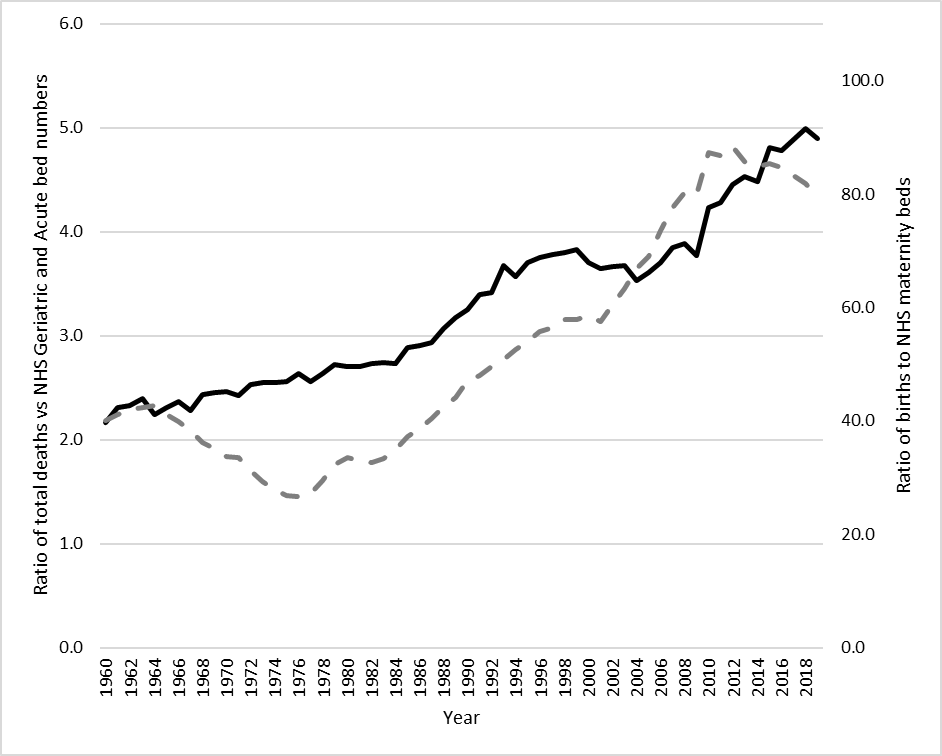

Supplement: S2 File — (DOCX) [file pone.0330931.s002.docx]
