# Supplementary material for: Long term trends in NHS inpatient bed provision in England, 1960–2020
Source: PLoS One. 2025 Aug 29;20(8):e0330931. doi: 10.1371/journal.pone.0330931 (PMC12396719; doi:10.1371/journal.pone.0330931)
Supplement: Attachment — Submitted filename: Response to reviewers 5.8.2025.docx [file pone.0330931.s005.docx]

Dear Professor Green,

Thank you for the opportunity to respond to the reviewers’ comments. We have set out the responses below in blue. A revised manuscript has been resubmitted, both a marked up copy with the changes highlighted in red and an unmarked copy.

**Reviewer #5**: This is an interesting and important research study. The main aim is to analyse how the number of beds changed over a 60-year period (1960-2020). The study will benefit if the authors can identify the factors (or explain the reasons) that are behind the reduction in the number of beds in the NHSE. So far, is too descriptive and does not provide information of the drivers of that bed reduction. Therefore, the authors need to clarify that they only find associations between variables rather than causal effects. We had added the following to limitations section to reflect this; “*Due to the nature of the analysis undertaken this paper only identifies associations and we have not demonstrated causation.”*

Most comments from previous review have been addressed. Though there are some remaining that need clarification. Also, I incorporate additional comments that would improve the manuscript and will ease understanding from readers outside the UK.

1. Comment 14 (Page 14 – “reduction in Geriatric bed capacity by three quarters, and even more if age is controlled for”. Where is this data?). The authors answered, “This is shown in Appendix 1.”.
• Is Appendix 1 Supplementary file 1? This is a Figure. Maybe they referred to Supplementary file 2 where there is a table showing “The number of five different categories of NHS beds and the provision of beds per 100,000 population in England in 1960 and 2019/20.” However, nothing is mentioned about controlling for age besides commenting that Geriatric figures only include 65+ patients and the other categories total population.

The reviewer is correct that the supplementary file 1 has been mislabelled. We apologise for the confusion. This has now been corrected in the text and the numbering of the supplementary files changed accordingly. In addition, Supplementary file 1 has been added to, with the population estimates for 1960 and 2019/20 now shown, both total population and population aged 65+.

2. Comment 16a (Over the years, the understanding and approach to treating and supporting individuals with learning disabilities have evolved significantly. How could these changes have had a direct impact on the provision and reduction of inpatient beds for this population? Would this explain all the bed reduction?). The authors replied, “The paragraph at the end of page 9 addresses this point and we have added the following: “The understanding and approaches to supporting people with Learning Disability has evolved significantly over the past 60 years, and could explain a significant proportion of the bed reductions seen here over the time period.””.
• I think the authors should make these comments if they are supported by the literature. Otherwise, it looks authors’ speculation with no evidence backup. I suggest that the authors include evidence that supports this. This can be explained by changes in the health policy by offering more services at the community level such as in the NHS Five Year Forward Review or the NHS Long Term Plan.

We have added the following and highlighted the most important of the NHS plans in this regard, *“These drivers to reduce Learning Disability bed numbers have also been incorporated into consecutive English policies to move from hospital based services to community delivered care such as the Five Year Forward View in 2014 , the 2019 NHS Long Term Plan , and most recently in the 2025 “Fit for the Future: 10 year plan for England”* (Pages 27 & 28).

3. Comment 17 (The authors state that there are two main limitations of the study: NHS England data only and lack of information on number of inpatient hospital beds. However, the authors only publish average number of hospital beds per year per category, which raises the question of what additional data could have been controlled for to enhance the study’s robustness. Did the authors not have access to additional data? If not, please state. Otherwise, important variables should be address, such as: socioeconomic factors, population density, urbanization, chronic disease prevalence, and healthcare access (e.g., number of healthcare providers per capita and availability of outpatient services) (…)). The authors responded “Data on Gross Domestic Product and annual change in this has been added to the analysis as well as the the number of deaths and births. We have also added data on Day Only beds, length of stay, the number of inpatient episodes of care, occupied bed days from 1998 onwards to help address the points raised. Reference has been added along with “including psychiatric beds” added to the introduction. The reference section has also been reviewed as suggested with five addition references added and some removed.”.
• I don´t think the authors incorporated the comments suggested in previous reviews. So far, the study is very descriptive with little information to understand the main drivers of this reduction in the number of beds. It could be understood that there was a disparity in the population growth and the number of available beds, but this is unclear. If the authors are using HES data, they can provide more information controlling for socioeconomic factors, for example. Also for Charlson co-morbidities. The authors should think about this question and answer the question addressed by reviewers.

We acknowledge this important limitation and have expanded the limitations section with the following, “*A limitation is the lack of information linking HES data and hospital bed numbers to socioeconomic measure for all but the most recent years. For example data on the amount of inpatient activity according to levels of deprivation was only available for the last three years of the 60 year period and was not available for any year for hospital bed numbers. Nor was there access to data linking hospital bed numbers or HES data with population density, chronic disease prevalence, or measures of comorbidity.”*

Additional comments:
4. The document provided includes a total of 31 pages. But from page 15, page numbers do not continue previous numbering. Can the authors revise this issue, please? The numbering has been addressed as requested and is now numbered pages 1 – 33.

5. There is a typo on page 3. It says COID19 and should say COVID-19. This has now been corrected but now appears on the first line of page 4.

6. On page 3 the authors should mention why the ratio of deaths to inpatient bed is important for international comparisons. A sentence has been added regarding this – “*this ratio helps to account for the ‘nearness to death effect’ on hospital bed use which significantly impacts demand and therefore provides a more accurate international comparisons than simply looking at beds per capita, as it acknowledges that a large proportion of hospital bed usage occurs in the last year of life*”.

7. Page 4 – Material and Methods – The authors mention that they included Day Only beds. Could you explain what kinds of procedures include this concept? I think they are linked to outpatient care (OPC) procedures, but I think this could be clarified, for example if you need to be admitted the day before or you can undergo the procedure in the same day and you will be discharge in few hours during the day. The following has been added – “*Day Only beds are located in wards that are open during the day but not overnight and typically are used for day surgery cases or other procedures that do not require overnight stay*”.

8. Page 5 – The authors mentioned on page 4 that they got the numbers of inpatient beds following a data request. However, they mention on page 5 that their main data source is HES. Can you clarify this? The main data source for hospital bed numbers was Hospital Activity Statistics on the NHS England website with data available from 2008/9. The data request was made for bed numbers prior to 2008. A sentence has been added on Page 4 at the start of the Materials and methods section to clarify this.

Also, which HES module you asked for APC, OPC, ACC,…? You can briefly describe each module in 1-2 sentences.

The HES data used was publicly available and was taken from Hospital Admitted Patient Care module which includes all episodes of care for admitted patients to hospital including days cases and critical care. It does not include out-patient appointments or accident & emergency attendance. Two sentences have been added, “*This module includes all episodes of care for patient admitted to hospital including day cases and critical care. It does not include out-patient appointments or accident and emergency appointments*.”

9. Page 5 – how many hospitals are you including? In England there are more than 200 NHS hospitals and some private providers. Since you are also discussing the participation of the private (independent) sector, it would be good to know the proportion in hospitals in England. The number of healthcare organisations providing services has been added in the first paragraph of page 6 along with the following sentence, “*In 1998/9 all this care was recorded as being provided by 348 NHS organisations. By 2019/20 some of this inpatient care was being provided by the Independent Sector (83 organisations) as well as by 237 NHS organisations. Many organisations in the NHS and Independent Sector operated more than one hospital but the precise number of hospitals is not available*”.

10. Page 6 – In the Statistical analysis section, the authors mention “The annual percentage change (APC) in bed provision was the variable used to measure rate of change”. Is this relative to population change or relative change to previous year? Both apply as it is the change from one year to the next calculated using the number of beds per 100,000 population in the same year. The following sentence has been added, “*This was calculated as the percentage change from one year to the next using population adjusted rates*”.

11. Page 7 – RCS & LOESS methods –These methods only provide information on potential associations between variables, not causal effects. Therefore, the authors need this to be clear. Moreover, it would be good to include a Supplementary material where the authors explain briefly how the RCS operates (i.e. using Lusa L, Ahlin Č. Restricted cubic splines for modelling periodic data. PloS One. 2020 Oct 28;15(10):e0241364. Doi: 10.1371/journal.pone.0241364). For the LOESS methods, I suggest the authors clarify this is a function that smooths fitted values. We have updated the second paragraph of the statistical analysis section to read as follows:

“RCS model non-linear relationships by fitting piecewise cubic polynomials between predefined knots. They divide the range of the data into sections using points called knots and fit smooth curves (cubic polynomials) between these sections. To prevent overfitting and improve interpretability, the model is ‘restricted’ (i.e., constrained) to be linear at the extremes (beyond the first and last knots). The number of knots for the RCS were obtained by fitting several spline models and comparing their AIC/BIC (Akaike Information Criterion / Bayesian Information Criterion), to determine which number of knots gave the best model fit. We also explored the suitability of other non-linear models including fractional polynomials, and non-parametric (LOESS), but RCS outperformed them. LOESS (Locally Estimated Scatterplot Smoothing) is a non-parametric regression technique that fits a smooth curve to a set of data points by using local weighted regression. Instead of assuming a single global function, LOESS fits many local regressions using weighted least squares, giving more influence to points near the target value, resulting in a smooth curve that closely follows the data trend without imposing a specific functional form”. These non-linear models help explore associations between the variables and outcome, but we do not use them to infer causality.”

12. Page 8 – there is a typo in the 3rd paragraph. Where it say “The largest reductions in numbers…”, then it should say “The largest reduction in the number of beds”. We agree that this should refer to number of beds and had already made this change but the strikethrough is not clear so have highlighted this in red. We think that as there was more than one category of beds that reductions is more accurate than reduction.

13. Page 8 – I suggest the authors move the paragraph above Day Only beds on page 9 to page 8 above the paragraph starting “At the start of the study Acute beds and Mental Illness beds (…)”.

We thank the reviewer for this suggestion and have moved the paragraph although it is now on page 9.

14. Page 9 – regarding Geriatric beds, what is the proportion of 65+ patients in the population and the sample? Please see response to point 1 above.

15. Page 11 – Figure 3 shows declining and accelerating periods. The accelerating bed closures in 1980-1990 was briefly explained in the Discussion (page 7 +15). What about the second period 2000-2010? I am sure there were changes in the provision of the service at community level but also health budget cuts that may contribute to this decision. Could you compare bed closures to population growth in those years?

We thank the reviewer for this comment. In response we have added the following paragraph regarding the second period of accelerated bed closures. “*The second period of accelerated bed closures followed on from the publication of the NHS Plan in 2000 that aimed to increase bed numbers and improve inpatient facilities by aiming to build 100 new hospitals, and encouraging public-private partnerships in the delivery of NHS funded care. However, we demonstrate that a second period of accelerated NHS bed closures followed*” (Page 26).

16. Page 12 – last paragraph. Please include a gap when you say, “11 million”. Are you saying that the 11% reduction in Occupied beds is explained by halving the average lengths of admissions? I think you wanted to say length of stay of admitted patients. Is this explained by new procedures/treatments or less severe diagnoses?

We thank the reviewed for pointing this out. A gap has been inserted, and we have clarified that the change in length of stay relates to “inpatient” admissions in the first paragraph of page 14. In the discussion on page 22 the following has been added regarding the reduction in length of stay “*in part related to new procedures and treatments*”.

17. Page 14 – what is the proportion of beds provided from the Independent (private) sector compared to the NHS provision?

These data are not publicly available for the period covered. The following has been added to the limitations. “*Data on the number of inpatient beds provided by the independent sector for the period covered were not available*.”

18. Page 1 (+15) – From Table 2 onwards, the authors should renumber continuing from page 15.

The numbering of pages has been changed.

19. Page 2 (+15) – Table 4 includes p-values. It is suggested that the table incorporates standard errors. The Table incorporates p-values and highlights coefficients that are statistically significant from the Poisson regression. Which variables were included in the regression model? Moreover, the authors may consider replacing p-values with stars, indicating the significance level in the coefficients (*0.05 significance level, **0.01 and ***0.001). That will simplify the understanding of the Table. Did the authors control for providers fixed effects to account for heterogeneity?

We have made the following changes:

1. Added std errors of the coefficients to the table.
2. Added significance level codes *, ** and *** for ease of interpretation and added a footnote to Table 4.

On whether “the authors control for providers fixed effects to account for heterogeneity?” – We fit Poisson mixed effects models and have region fixed effects to account for the heterogeneity (as specified in methods section). In this work, the region fixed effects enable us to account for the heterogeneity in bed numbers. We do not have individual provider bed numbers data within each region to account for the more granular variability, but assume that there is reasonable homogeneity across providers within each region.

20. Page 3 (+15) – There is a typo in the first paragraph. Where it says, “This ratio was much higher in London (440, N=1) then…”. This “then” should be replaced with “than”.

This has been corrected.

21. Page 7 (+15) – As I mentioned in Comment 15, please discuss the second period of accelerated bed closures 2000-2010. Please see response to comment 15 above.

22. Page 11 (+15) – Strengths and limitations. I am not sure if a 60-year period is a strength. In such long period there is too much heterogeneity caused by different economic cycles and changes in regulation. I would say that using HES data is a strength if the authors justify this rich data with analyses incorporating demographics and other relevant socioeconomic controls. But the authors should make clear if they are using HES in their analysis. So far, it is not clear. Regarding demographics, the Results have been added to regarding changes in gender and age that is available on HES from 1998/99 as follows, “*In 1998/9, the average age of patients was 45 years and 57% were recorded as female gender. By 2019/20 average age had increased to 54 years, and 55% were recorded as female. The largest increase in episodes of care was in the elderly with a 147% increase for those aged 75+ years (2.2 to 5.5 million FCE) compared with 20% increase in those aged 0-14 years (1.7 to 2.0 million FCEs).”*

Unfortunately, the amount of information linking HES with socioeconomic information is limited. For example data linking admissions with deprivation and with ethnicity was only available for the last 3 of the 60 years (from 2017/8). The strengths and limitations section has been added as follows, “*the 60-year period can also be considered a limitation due to the different economic cycles and several changes in NHS regulation that occurred during this time period. The use of Hospital Episode HES) Statistics in addition to Hospital Activity Statistics is another strength as the former measures inpatient activity whilst the later measures inpatient bed numbers. In addition from 1998/99 demographic information is available including age and gender for HES.*

*A limitation is the lack of information linking HES data to socioeconomic measure for all but the most recent years. For example, data on the amount of inpatient activity according to levels of deprivation of residence was only available for the last three years of the 60-year period and was not available for any year for hospital bed numbers. Nor did we have access to data linking HES activity to population density, chronic disease prevalence or measures of co-morbidity*.”

**Reviewer #4:** This paper examines trends in inpatient beds in the UK from 1960 to 2020. This is the first revision of the manuscript, but I can’t see the “responses to reviewers”. However, based on the revised and track-changed version of the manuscripts, my comments/questions for the paper are:

1. What is the rationale/hypothesis to test for a trend in NHS inpatient bed provision in England in 1960-2010? (e.g., technological progress, policy changes, demographic shifts)? The null hypothesis been more explicitly stated as “to test the null hypothesis that the annual reduction in NHS inpatient beds occurred uniformly across the 60 year period and affected all sectors/bed types equally”

2. What are the results of detailed analysis (e.g., restricted cubic splines, fractional polynomials, LOESS?

We only present results of the restricted cubic spline as it outperformed LOESS and factional polynomials. We do not present any results of the LOESS or fractional polynomials as these were explored alongside RCS to determine which gives the best fit. The main output from the RCS fit is the visualisation in Figure 3, which is our predicted RCS model overlaid on actual values (raw data). We have appended model output in supplementary file 2.

3. What variables did the authors use to control for factors affecting the trend (e.g., changes in reporting standard, LOS, population structure), what are the estimated parameters of those factors? We used data that was consistently reported across the 60-year period where possible. There was some data that was only available from 1998/1999 and this is described in the methods section. Information presented on the change in the population aged 65+ that occurred during the study period and the rate of Geriatric bed provision is calculated according to this. See response to point 1 from reviewer 5.

4. The authors have a long time series data of 60 years but it looks like they estimated the average of arithmetic mean of percentage changes of each year. This approach can be useful for understanding short-term fluctuations, but it does not accurately reflect long-term growth trends. This long-term trend should be estimated by taking the logarithm of the outcome (inpatient beds) and regressing it over the time variables (year). We thank the reviewer for this suggestion. We agree with the reviewer that modelling the outcome (or any transformation of it like log) against the time variables is a useful approach. This is exactly what we have done, when we fitted a Poisson model to capture those variabilities. However, we’d also like to differ to some degree with their proposal in the following ways:

1. A logarithmic transformation is dependent on whether we need one or not.
2. We do not “estimated the average of arithmetic mean of percentage changes of each year”. Instead, the model estimates those predicted percentage changes on average, or what they would be on average (essentially what you get from a model). This is NOT “the average of..” as suggested by the reviewer.
3. Importantly, statistically, modelling of this type can be approached in two ways which lead to the same conclusions. 1) Either we model the outcome and regress it against the time variables, OR, 2) model the percentage changes in outcome over time, and regress that against time variables but including the baseline value as a covariate. This way, models 1 and 2 achieve the same conclusions and similar inferences may be drawn.
4. Based on the immediate above, we model percentage changes because it is easy to make inferences from. Given the decline in numbers over time, we are more interested in the rate of change of the evident downward trend.

We hope these responses along with the changes made to the manuscript adequately address the reviewers comments. Thank you for your further consideration of our paper for publication in PLOS One.

Yours sincerely,

Patrick Keown on behalf of the authors.

August 2025
